# Etoricoxib Coated Tablets: Bioequivalence Assessment between Two Formulations Administered under Fasting Conditions

**DOI:** 10.3390/pharmaceutics15112569

**Published:** 2023-11-01

**Authors:** Jessica Meulman, Marcelo Gomes Davanço, Débora Renz Barreto Vianna, Thalita Martins da Silva, Fernando Costa, Fernando Bastos Canton Pacheco, Milla Emke de Oliveira, Celso Francisco Pimentel Vespasiano

**Affiliations:** 1 Clinical Research Unit, Medical Department, Adium S.A., São Paulo 04794-000, SP, Brazil; marcelo.davanco@adium.com.br (M.G.D.); debora.vianna@adium.com.br (D.R.B.V.); thalita.silva@adium.com.br (T.M.d.S.); celso.vespasiano@adium.com.br (C.F.P.V.); 2 Clinical Studies Management, Research and Development Department, Monte Verde S.A., Munro, Buenos Aires B1605EBQ, Argentina; fcosta@raffo.com.ar; 3 Bioequivalence Unit, Centro Avançado de Estudos e Pesquisas Ltd., Campinas 13087-567, SP, Brazil; fernando.pacheco@synvia.com (F.B.C.P.); milla.oliveira@synvia.com (M.E.d.O.)

**Keywords:** etoricoxib, anti-inflammatory agents, non-steroidal, cyclooxygenase 2 inhibitors, bioequivalence, pharmacokinetics, safety

## Abstract

Etoricoxib is a non-steroidal anti-inflammatory drug with high selectivity for cyclooxygenase 2 (COX-2), exerting a pronounced anti-inflammatory effect with fewer adverse events when compared to COX-1 inhibitors. The present study aimed to evaluate the bioequivalence between two etoricoxib-coated tablet formulations to meet regulatory requirements for a branded generic product registration in Brazil. A crossover study with an open-label, randomized design and a single-dose regimen with two treatments and two periods was conducted on healthy Brazilians of both genders. Subjects randomly received a single dose of a 90 mg etoricoxib coated tablet of test product Xumer^®^ 90 mg (Adium S.A.) and the reference product Arcoxia^®^ 90 mg (Merck Sharp & Dohme Farmacêutica Ltda.) under fasting conditions separated by a 14-day period. Blood samples were collected sequentially for up to 96 h following drug administration, and the concentrations of etoricoxib in plasma were determined using a validated UPLC-MS/MS method. Pharmacokinetic parameters were computed utilizing non-compartmental analysis methods. A total of 32 healthy subjects were enrolled, and 25 subjects completed the study. Geometric mean ratios (90% confidence intervals) for C_max_, AUC_0-t_, and AUC_0-inf_ were 103.98% (95.63–113.06), 96.82% (91.82–102.09), and 95.79% (90.70–101.16), respectively. In accordance with regulatory standards, the test formulation (Xumer^®^ 90 mg) has been deemed bioequivalent to the reference product (Arcoxia^®^ 90 mg). As a result, these formulations can be considered interchangeable in clinical practice, with both proving to be safe and well-tolerated. The need for in vivo testing for the Xumer^®^ 60 mg strength was waived due to the proportional similarity of the formulations and the similar in vitro dissolution profiles observed across the various strengths.

## 1. Introduction

When it comes to the management of acute and chronic pain conditions, non-steroidal anti-inflammatory drugs (NSAIDs) represent the most frequently prescribed therapeutic choices. These agents hinder the production of prostaglandins by blocking the action of cyclooxygenase (COX), which results in decreased pain and inflammation, ultimately providing relief and analgesia [1,2,3]. COX-1 is an enzyme found in nearly all tissues, including blood vessels, platelets, stomach, intestine, and kidneys, and is referred to as a constitutive enzyme [4,5]. One of its essential functions is to produce prostaglandins, which have significant physiological effects that include protecting the gastric lining, promoting platelet aggregation, maintaining vascular balance, and regulating blood flow in the kidneys; hence, the inhibition of COX-1 can lead to gastric damage, hemorrhage, and ulceration [2].

COX-2, conversely, is classified as an inductive enzyme due to its predominant presence at inflammatory sites. It is primarily produced by cells involved in the inflammatory cascade, including macrophages, monocytes, and synoviocytes. Additionally, COX-2 has been detected in several other tissues and organs, including the kidneys, brain, ovaries, uterus, cartilage, bones, and vascular endothelium [4,5]. The inhibition of COX-2 provides a unique opportunity to block the production of inflammatory prostaglandins while sparing the prostaglandins generated by COX-1 in the stomach, so the primary goal of COX-2 inhibitors is to reduce adverse effects, preserving beneficial therapeutic effects [1,4,5].

Etoricoxib is an NSAID highly selective for COX-2, with a 106-fold estimated selectivity [1,6,7]. In comparison with other agents of this class, such as rofecoxib, valdecoxib, celecoxib, meloxicam, naproxen, and ibuprofen, in human whole blood, etoricoxib displayed greater selectivity for COX-2, as evidenced by higher IC_50_ ratios for its inhibition in comparison to the other agents. In Brazil, etoricoxib is currently indicated for acute and chronic treatment of the signs and symptoms of osteoarthritis and rheumatoid arthritis, for the treatment of ankylosing spondylitis, relief of chronic and acute pain, treatment of moderate to severe post-surgical pain associated with dental surgery, and for the treatment of moderate to severe acute post-surgical pain associated with abdominal gynecological surgery [8].

After oral administration, etoricoxib is well absorbed and presents a mean oral bioavailability of approximately 100%, with peak plasma concentrations at around 1 hour after the intake [8,9]. Etoricoxib has a high degree of protein binding, around 92%, and possesses a steady-state volume of distribution (Vdss) of approximately 120 L [8]. Metabolism plays a significant role in the transformation of etoricoxib, as less than 1% of the administered dose is excreted unchanged in the urine. The primary metabolic route leading to the creation of the 6′-hydroxymethyl metabolite is facilitated by cytochrome P450 (CYP) enzymes, mainly through CYP3A4 isoenzyme [8,9]. Steady-state concentrations of etoricoxib are attained after 7 days of once-daily administration at a dose of 120 mg. In this equilibrium state, there is an accumulation ratio of approximately two, corresponding to an accumulation half-life of around 22 h. The estimated plasma clearance stands at approximately 50 mL/min. It is worth noting that etoricoxib’s pharmacokinetics maintain linearity within the clinically relevant dose range [8,9].

Bioequivalence studies are performed to evaluate if different formulations with the same active pharmaceutical ingredient and studied under the same experimental design present equivalent bioavailability. The evaluation of test and reference formulations includes an assessment of their pharmacokinetic profiles and a bioequivalence analysis, with the aim of confirming that both products share the same rate and extent of absorption [10]. The primary objective of this study was to evaluate the bioequivalence and tolerability of two formulations of 90 mg etoricoxib-coated tablets. This evaluation was conducted to satisfy the regulatory criteria set by the Brazilian National Health Surveillance Agency (ANVISA) for the registration of branded generic products [10].

## 2. Materials and Methods

### 2.1. Study Population

All the phases of the bioequivalence study were conducted at Centro Avançado de Estudos e Pesquisas Ltda. (CAEP), a CRO certified by ANVISA located in Campinas, São Paulo, Brazil. The research protocol underwent evaluation and received approval from Investiga—Institutos de Pesquisa Ethics Committee under protocol number 4.460.173. The study was conducted in strict adherence to established guidelines, including the Good Clinical Practices Guidelines [11], the ethical principles for medical research involving human subjects as outlined in the Declaration of Helsinki [12], the current Brazilian ethical legislation (Resolution number 466/2012, Ministério da Saúde) [13] and ANVISA’s requirements for bioequivalence studies. Prior to the initiation of study procedures, informed consent was appropriately secured from all participants. Biological data and materials were used only for the specific purposes of the present study, maintaining the confidentiality of the data.

A group of 32 healthy individuals was selected, comprising 16 men and 16 women, with ages ranging from 18 to 50 years and a body mass index (BMI) falling within the range of 18.5 to 29.9 kg/m^2^. Among the inclusion criteria are to be in good health conditions or have no significant illness identified at the researcher’s discretion or through assessments such as clinical history, electrocardiograms, vital signs, physical examinations, anthropometric measurements, and laboratory tests; to present a negative test for COVID-19 (as it was conducted during the COVID-19 pandemic); to comprehend the study’s objectives, its nature, associated risks, and potential adverse events; to adhere to the study protocol, as affirmed by signing the Informed Consent Form (ICF); and to consent to the use of a reliable contraceptive method.

### 2.2. Drug Products

The test product was Xumer^®^ (etoricoxib) 90 mg coated tablet (batch No. 86663, expiry date: January 2022) manufactured by Monte Verde S.A. (Pocito, San Juan Province, Argentina) and imported by Adium S.A. (Pindamonhangaba, São Paulo, Brazil), and the reference product was Arcoxia^®^ (etoricoxib) 90 mg coated tablet (batch No. S038471, expiry date: July 2022) manufactured by Frosst Ibérica S.A. (Madrid, Spain) and imported by Merck Sharp & Dohme Farmacêutica Ltda (Campinas, São Paulo, Brazil). In vitro tests were carried out before the start of the clinical phase with the batches mentioned above, which confirmed the in vitro pharmaceutical equivalence of both products.

### 2.3. Study Design

The present bioequivalence study, conducted as a single-center trial, was designed as an open-label, randomized, and crossover study with two treatment periods and two sequences. In each period, participants received either the test or the reference product under fasting conditions, with a 14-day washout period. In this design, the balance between genders was taken into account due to the participation of both male and female subjects. Therefore, stratified randomization with blocks was applied, in which each block (subject) received the two formulations in different periods, with sequences randomly assigned and in a balanced way to minimize sequence and period effects.

In each study period, participants underwent a minimum overnight fasting period of 8 h before receiving a single oral dose of 90 mg etoricoxib along with 200 mL of water. They remained in a fasting state for 4 h following drug administration, and no liquids were permitted within 2 h prior to and 2 h after taking the medication. In order to ensure uniformity among treatment groups, all subjects in both periods adhered to an identical standard diet, including food and beverages. The consumption of alcoholic beverages, as well as foods or drinks containing caffeine or xanthine (such as coffee, chocolate, tea, and cola- or guarana-infused soft drinks), was strictly prohibited. A total of 22 blood samples were collected at 0 h (1 h before drug administration) and 0.25, 0.50, 0.75, 1.00, 1.33, 1.67, 2.00, 2.33, 2.67, 3.00, 3.50, 4.00, 5.00, 6.00, 8.00, 10.00, 12.00, 24.00, 48.00, 72.00 and 96.00 h after drug administration. Following collection, the blood samples were promptly subjected to centrifugation for 10 min at 4 °C at 3500 rpm (equivalent to 2301 g). The resulting plasma was then carefully separated, placed into amber cryogenic tubes, appropriately labeled, and subsequently stored at −70 °C until it was ready for analysis. The primary outcome was the assessment of the bioequivalence of the test and reference product through the pharmacokinetic parameters C_max_ (maximum plasma drug concentration) and AUC_0–t_ (area under the curve from zero to the last quantifiable concentration). Secondary outcomes were evaluation and descriptive analysis of pharmacokinetic parameters AUC_0–inf_ (area under the curve from zero to infinity), T_max_ (time of the occurrence of C_max_), t_1/2_ (elimination half-life), kel (elimination constant), Vd (volume of distribution) and Cl (clearance) of test and reference drugs, and also evaluation of safety and tolerability through reporting of adverse events.

### 2.4. Bioanalytical Method

Etoricoxib plasma concentrations were quantified using a validated UPLC-MS/MS method, which involves reversed-phase chromatography coupled with mass spectrometry. The system includes a UPLC (Waters UPLC, Milford, MA, USA) and a XEVO TQ-S (Waters, Milford, MA, USA). Etoricoxibe-d6 was used as an internal standard (IS), and the extraction was performed through protein precipitation using acetonitrile.

A volume of 0.4 µL from each sample was introduced into a C18 column (Waters Acquity UPLC BEH C18, 2.1 × 50 mm) maintained at a temperature of 40 °C. The mobile phase was composed of a blend of ammonium formate (20 mM) and acetonitrile in a 1:1 ratio (*v*/*v*). The flow rate was set at 0.3 mL/min, employing a gradient approach. To minimize variations between measurements, all samples from each participant were analyzed together in a single analytical run. For mass spectrometry detection, an electrospray ionization source in positive mode was utilized. The analysis employed a multiple reaction monitoring (MRM) method, where specific transitions were monitored for etoricoxib and IS at *m*/*z* 359.1 → 280.1 and *m*/*z* 365.1 → 286.1, respectively. To determine the concentrations of the analyte in the subject samples, interpolation on the calibration curve was applied. The calibration curve was designed with a linearity range spanning from 10 to 4500 ng/mL.

The bioanalytical method underwent comprehensive validation following the guidelines provided by ANVISA for bioanalytical method validation [14], in which the acceptance criteria for selectivity and concomitant selectivity tests are that the responses of interfering peaks close to analyte retention time must be lower than 20% of the analyte response in Lower Limit of Quantification (LLOQ) samples and the responses of interfering peaks close to PI retention time must be lower than 5% of the IS response, the same is considered for the carry-over effect. For the matrix effect, the coefficient of variation (CV) of internal standard normalized matrix factor (ISNMF) related to all samples must be lower than 15%. For the approval of calibration standards, deviation must be smaller than or equal to 20% compared to the nominal concentration for LLQ patterns; and smaller than or equal to 15% compared to other calibration standards. The acceptance criteria for the calibration curves are to have minimum 75% of the calibration standards approved according to the previous criteria and at least 6 calibration standards of different concentrations, including LLQ and upper limit of quantification (ULOQ). For precision and accuracy, coefficient of variation values above 15% are not permitted, except for LIQ, for which values less than or equal to 20% are accepted. For stability tests, the acceptance criteria is to not present deviation higher than 15% of the average concentrations obtained regarding the nominal value.

### 2.5. Statistical Analysis

The sample size was determined based on previously published data of intra-subject variability for C_max_ of etoricoxib around 21%, expected difference between treatments of 5%, significance level (α) of 5%, power of 80%, and also taking into account possible drop-outs and exclusions in order to guarantee the reliability of the statistical results.

Pharmacokinetic parameters were derived from the plasma concentration–time curves of etoricoxib and then statistically compared to assess bioequivalence using the R^©^ software. AUC_0–t_ was computed using the trapezoidal method, AUC_0–inf_ as AUC_0–t_ + (Cn/kel), where Cn represented the last quantifiable plasma concentration. The elimination constant was derived from the elimination phase of the graph, where log plasma concentration was plotted against time. The elimination half-life was calculated using the equation t_1/2_ = Ln(2)/kel. The volume of distribution was calculated through the ratio between the amount of drug in the body and its concentration in the biological matrix, and clearance was determined by multiplying the volume of distribution and the elimination constant. Maximum plasma drug concentration and the time to reach it (T_max_) were directly extracted from the experimental data. The assessment of bioequivalence relied on predefined acceptance criteria in accordance with ANVISA requirements [10]. If the extreme values of the 90% confidence interval of geometric means ratio (AUC_0-t_ test/AUC_0-t_ reference and C_max_ test/C_max_ reference) are greater than 0.80 and less than 1.25, products can be considered bioequivalent. To evaluate the sequence, treatment, and period effects, an ANOVA test was conducted on the parameters C_max_, AUC_0–t_, and AUC_0–inf_. This analysis allowed for a comprehensive examination of potential variations related to the sequence of administration, the treatment received, and the specific period under consideration.

### 2.6. Safety

Throughout this study, continuous monitoring was applied to all participants to ensure their safety. The safety assessment encompassed the continuous monitoring of vital signs, which included temperature, blood pressure, and heart rate. These measurements were taken both at the baseline before dosing and at various points throughout the study. Furthermore, comprehensive evaluations were conducted, such as laboratory assessments covering hematology, urinalysis, and blood biochemistry, in addition to physical examinations and electrocardiograms (ECGs) that were performed at the study’s initiation and conclusion.

To detect and address any potential adverse events, participants were observed closely throughout the entire study period and were informed about the importance of promptly reporting any unfavorable symptoms or medical conditions they experienced during the study or following hospitalization. In cases where adverse events occurred, they were categorized according to their severity, falling into classifications of mild, moderate, or intense. The medical staff then determined the causality of these events to the drug, using criteria such as defined, likely, possible, unlikely, conditional/unclassified, or not accessible/not classifiable. This comprehensive approach ensured a thorough evaluation of the participants’ safety throughout the study period.

## 3. Results

The objective of this study was to assess if the test product is bioequivalent to the reference product when administered to fasting participants. To achieve this, an open-label, randomized, single-dose, crossover bioequivalence study was successfully performed. The study was carefully balanced between genders, and a 14-day washout period separated the two study periods.

### 3.1. Study Population

Thirty-two healthy individuals were initially enrolled in the study, but ultimately, only twenty-five participants (comprising 13 women and 12 men) successfully completed both study periods and were consequently included in the subsequent pharmacokinetic analysis. Since the study was conducted during the COVID-19 pandemic, four participants were excluded for not showing up for the RT-PCR test for COVID-19 before the second period of hospitalization. One participant was excluded for personal reasons, and two due to absence or significant delays in blood collections. Table 1 provides a description of the demographic characteristics of the study participants.

### 3.2. Bioanalytical Method

The bioanalytical method underwent a comprehensive validation, encompassing all essential assays, such as selectivity and concomitant selectivity, carry-over effect, calibration curve, precision, accuracy, matrix effect, and stability assessments. The selectivity of the method was confirmed to be adequate, as no interference from substances in the blank plasma samples was observed during the retention times of etoricoxib and IS, showing an LLOQ of 10 ng/ml. The absence of etoricoxib detection in the pre-dose plasma samples of any participant indicated the absence of carry-over effects and validated an adequate washout period. The calibration curve was linear in the range of 10.0 to 4500.0 ng/mL. Regarding precision and accuracy, the method proved to be suitable for samples prepared within the same assay (intra-run) as well as across different assays (inter-run), ensuring reliable and consistent results. Stability tests indicated that the samples exhibited stability at room temperature (+15 °C to +25 °C) for as long as 18 h and for up to 139 h after extraction. Additionally, the samples maintained their stability even after undergoing five freezing and thawing cycles in both a standard freezer (−20 °C) and an ultrafreezer (−70 °C). Furthermore, these samples remained stable for an extended period of up to 143 days in both types of freezers. These stability assessments hold significant importance in ensuring the proper storage of samples until analysis, thereby ensuring the accurate determination of drug concentrations.

In summary, all validation parameters met the pre-established acceptance criteria in accordance with ANVISA bioanalytical method validation guidelines [14], confirming the reliability and suitability of the method for determining etoricoxib plasma concentrations.

### 3.3. Pharmacokinetic Analysis

**Figure 1:**
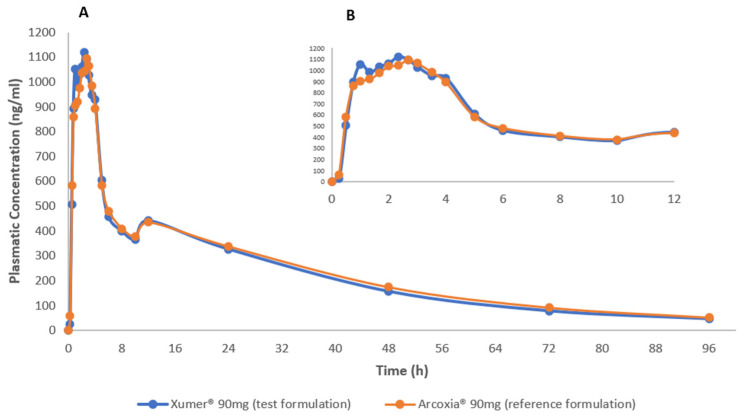
Mean etoricoxib plasma concentration vs time curves after administration of test (Xumer^®^) and reference (Arcoxia^®^) formulations in healthy subjects (male and non-pregnant female) under fasting conditions. (**A**) Plasma concentration from 0 to 96 h; (**B**) Plasma concentration vs time curves focused from 0 to 12 h.

Among the enrolled participants, twenty-five concluded both study periods and were included in pharmacokinetic analysis. Figure 1 displays the mean plasma concentration versus time curves for both the reference and test products. A notable observation is the remarkable similarity between the pharmacokinetic profiles of the two products. Additionally, the selected sampling time was found to be adequate, effectively capturing and describing the drug’s absorption and elimination phases.

Table 2 provides a description of the pharmacokinetic parameters obtained for both the test and reference formulations.

**Figure 2:**
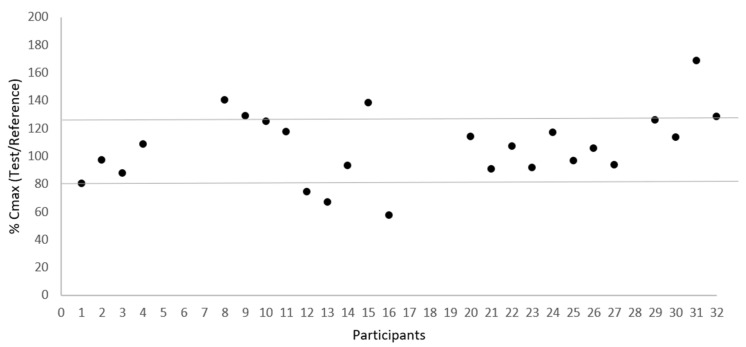
Dispersion of test/reference ratio for C_max_ between subjects (N = 25).

**Figure 3:**
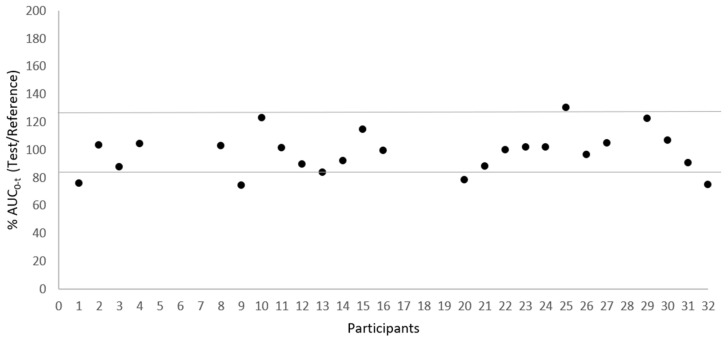
Dispersion of test/reference ratio for AUC_0-t_ between subjects (N = 25).

Figure 2 and Figure 3 illustrate the low variability in test/reference ratios for C_max_ and AUC_0–t_, respectively, among the 25 subjects who completed the study. These figures illustrate the uniformity of the data acquired in this investigation, showing a small variability within subjects between the test and reference for C_max_ and AUC_0–t_ ratios. This consistency is supported by the within-subject coefficient of variation (CV_ws_) values, which stand at 17.38% for C_max_ and 10.96% for AUC0-t, as presented in Table 3.

### 3.4. Bioequivalence Assessment

Table 3 describes the test/reference geometric mean ratios obtained for the pharmacokinetic parameters C_max_, AUC_0–t_, and AUC_0–inf_, along with the corresponding 90% confidence intervals (Cis), CV_ws_, and power for the bioequivalence analysis.

The 90% CI of logarithmically Ln-transformed the ratios (Test/Reference) of C_max_, AUC_0–t_, and AUC_0–inf_ were 95.63–113.06%, 91.82–102.09% and 90.70–101.16%, respectively, which were within the acceptable range of 80.00–125.00% established by ANVISA [10]. The *p*-values obtained for the fixed effects of ANOVA (Partial) showed that the sequence effect was not significant for the pharmacokinetic parameter C_max_ (*p*-value = 0.160), as well as for AUC_0–t_ (*p*-value = 0.644), at a 10% significance level. The treatment effect was not significant for C_max_ (*p*-value = 0.432) and for AUC_0–t_ (*p*-value = 0.307) as well, at a 5% significance level. The period effect was also not significant for C_max_ (*p*-value = 0.276) and for AUC_0–t_ (*p*-value = 0.545), at a 5% significance level.

### 3.5. Safety

A total of eight adverse events were reported by seven participants during hospitalization, and three adverse events were reported by two participants in the post-study period. Among the 32 participants of the safety population, headache emerged as the most incident adverse event, being reported by four (12.5%) participants. It is noteworthy that both test and reference drug groups experienced this adverse event, which is consistent with the reference drug’s prescribing information, which lists a headache as a common adverse reaction. The other reported adverse events presented the same incidence, as described in Table 4. No serious adverse events occurred during the study, and there were no reported pregnancies detected among the participants. All the adverse events were classified as of mild intensity.

## 4. Discussion

The results obtained in the present study are consistent with data available in the literature for etoricoxib-coated tablets. Shohag et al. [15] performed a single-dose, randomized, open-label, randomized crossover design with two periods, administering etoricoxib-coated tablets 60 mg with a two-week washout period and with blood collection up to 120 h post-drug administration. The values obtained for the pharmacokinetic parameters of the reference product were 1290 ng/mL for C_max_; 33,400 ng/mL·h for AUC_0–120_; 33,000 ng/mL·h for AUC_0–inf_; 2.63 h for T*max*; and 29.84 h for t_1/2_. No adverse effects were identified or reported in this study.

Suyatna et al. [16] performed an open-label, randomized, single-dose, two-period, two-treatment, crossover study with 24 healthy adult subjects, administering etoricoxib 90 mg coated tablets under fasting conditions with a washout period of 7 days and blood collection up to 96 h. The values obtained for the pharmacokinetic parameters of the reference product (Arcoxia^®^ 60 mg) were 1925.97 ng/mL for C_max_, 33,359.65 ng/mL·h for AUC_0–96_; 35,528.75 ng/mL·h for AUC_0–inf_; 1.00 h for T*max* and 19.89 h for t_1/2_ which are very close to our findings. Etoricoxib showed a positive safety profile since there were no adverse events reported in this study as well. A similar study was performed by Harikrishnan et al. [17] in 26 healthy male participants, using Arcoxia^®^ 120 mg as the reference product. The values obtained for the pharmacokinetic parameters were 2625.19 for C_max_, 48,028.655 ng/mL·h for AUC_0–72_, and 1.16 h for T*max*. Even using twice the dosage (120 mg), etoricoxib was shown to be safe and well tolerated. In this study, among the study participants, only two adverse events were reported, fever and headache, which were classified as mild intensity.

The 90% confidence intervals (CIs) obtained for the ratio of geometric means (test/reference) of logarithmically transformed pharmacokinetic parameters 95.63–113.06% for C_max_, 91.82–102.09% for AUC_0–t_, and 90.70–101.16% for AUC_0–inf_, were all within the predetermined range of 80.00–125.00%, with an adequate statistical power to detect a difference between treatments. The ANOVA test performed showed that there were no significant effects of sequence, treatment, and period in this analysis. The CVws obtained was 17.38% for C_max_, an intra-subject variability smaller than observed in the literature, showing that the sample size calculation was adequate for the purpose of the study.

In accordance with ANVISA requirements for biowaiver of additional strengths [18], further in vivo testing for Xumer^®^ 60 mg was not deemed necessary. This decision was based on the acceptance of a bioequivalence study conducted on the 90 mg strength, the proportional similarity of formulations across all strengths, comparable in vitro dissolution profiles for all strengths (specific data not presented), and the linear pharmacokinetics of etoricoxib across the 60 to 90 mg range.

## 5. Conclusions

In the present study, it was possible to successfully characterize the pharmacokinetic profile of both test and reference formulations through the determination of the pharmacokinetic parameters C_max_, AUC_0–t_, AUC_0–inf_, T*max*, and t_1/2_; kel, Vd, and Cl. Both formulations were well tolerated and showed similar safety profiles between them.

The 90% confidence intervals (CIs) obtained for the ratio of geometric means (test/reference) of logarithmically transformed pharmacokinetic parameters C_max_ and AUC_0–t_ were within the predetermined range of 80.00–125.00% required by ANVISA. Therefore, it can be concluded that the test formulation Xumer^®^ coated tablet is bioequivalent to the reference formulation Arcoxia^®^ in terms of rate and extent of absorption. Both formulations are expected to produce the same therapeutic response, making these products interchangeable in medical practice due to their identical efficacy and safety profiles.

## Figures and Tables

**Table 1:** Demographic characteristics of study participants.

Characteristic	Descriptive Statistics *n* = 32
**Age (years)**	.
Mean _(±SD)_	35.96 _(±8.99)_
Range	21–50
**Weight (kg)**	
Mean _(±SD)_	73.3 _(±10.3)_
Range	51.6–94.0
**Height (m)**	
Mean _(±SD)_	1.67 _(±0.08)_
Range	1.52–1.82
**BMI (kg/m^2^)**	
Mean _(±SD)_	26.09 _(±2.71)_
Range	20.57–29.90
**Sex (n [%])**	
Male	12 (48%)
Female	13 (52%)

**Table 2:** Pharmacokinetic parameters of test (Xumer^®^) and reference (Arcoxia^®^) formulations, administered under fasting conditions in healthy subjects (*n* = 25). Data expressed as mean _(±SD)_.

Parameter	Xumer^®^ (Test Formulation)	Arcoxia^®^ (Reference Formulation)
C_max_ (ng/mL)	1504.43 _(±99.46)_	1421.53 _(±71.12)_
AUC_0–t_ (ng/mL·h)	21,992.05 _(±1086.34)_	22,760.18 _(±1239.02)_
AUC_0–inf_ (ng/mL·h)	23,839.13 _(±1274.60)_	24,975.42 _(±1472.10)_
T_max_ (h)	2.03 _(±0.45)_	1.84 _(±0.19)_
t_1/2_ (h)	25.55 _(±0.95)_	26.58 _(±1.32)_
kel (1/h)	0.03 _(±0.001)_	0.03 _(±0.001)_
Vd (L)	145.32 _(±6.89)_	144.25 _(±7.07)_
Cl (L/h)	4.02 _(±0.20)_	3.86 _(±0.19)_

C_max_, maximum plasma concentration; AUC_0–t_, area under the concentration–time curve from zero to 96 h; AUC_0–inf_, area under the concentration–time curve extrapolated to infinity; Tmax, time to reach C_max_; t_1/2_, elimination half-life; kel, elimination constant.; Vd, volume of distribution; Cl, Clearence.

**Table 3:** Geometric mean ratio, confidence intervals (90%), CV_ws_ and power (N = 25).

Parameter *	Geometric Mean Ratio (%)	90% CI (%)	CV_ws_	Power (%)
C_max_	103.98	95.63–113.06	17.38	99.82
AUC_0–t_	96.82	91.82–102.09	10.96	100.00
AUC_0–inf_	95.79	90.70–101.16	11.29	100.00

* Parameters logarithmically transformed.

**Table 4:** Incidence of adverse events (N = 32).

Adverse Event	Incidence N (%)	Causality	Intensity
Headache	4 (12.5%)	Likely	Mild
Malaise	1 (3.1%)	Possible	Mild
Loose stools	1 (3.1%)	Likely	Mild
Colic	1 (3.1%)	Possible	Mild
Hypertension	1 (3.1%)	Unlikely	Mild
Hematuria	1 (3.1%)	Possible	Mild
Increased ALT	1 (3.1%)	Likely	Mild
Increased AST	1 (3.1%)	Likely	Mild

ALT: Alanine transaminase; AST: Aspartate transaminase.

## Data Availability

The data are not accessible to the public for confidentiality reasons.
